# Machine Learning-Based HIV Risk Estimation Using Incidence Rate Ratios

**DOI:** 10.3389/frph.2021.756405

**Published:** 2021-12-02

**Authors:** Oliver Haas, Andreas Maier, Eva Rothgang

**Affiliations:** ^1^Department of Industrial Engineering and Health, Institute of Medical Engineering, Technical University Amberg-Weiden, Weiden, Germany; ^2^Pattern Recognition Lab, Department of Computer Science, Technical Faculty, Friedrich-Alexander University, Erlangen, Germany

**Keywords:** HIV, risk estimation, association rules, bias, clinical data, machine learning, artificial intelligence, incidence rate ratio

## Abstract

HIV/AIDS is an ongoing global pandemic, with an estimated 39 million infected worldwide. Early detection is anticipated to help improve outcomes and prevent further infections. Point-of-care diagnostics make HIV/AIDS diagnoses available both earlier and to a broader population. Wide-spread and automated HIV risk estimation can offer objective guidance. This supports providers in making an informed decision when considering patients with high HIV risk for HIV testing or pre-exposure prophylaxis (PrEP). We propose a novel machine learning method that allows providers to use the data from a patient's previous stays at the clinic to estimate their HIV risk. All features available in the clinical data are considered, making the set of features objective and independent of expert opinions. The proposed method builds on association rules that are derived from the data. The incidence rate ratio (IRR) is determined for each rule. Given a new patient, the mean IRR of all applicable rules is used to estimate their HIV risk. The method was tested and validated on the publicly available clinical database MIMIC-IV, which consists of around 525,000 hospital stays that included a stay at the intensive care unit or emergency department. We evaluated the method using the area under the receiver operating characteristic curve (AUC). The best performance with an AUC of 0.88 was achieved with a model consisting of 53 rules. A threshold value of 0.66 leads to a sensitivity of 98% and a specificity of 53%. The rules were grouped into drug abuse, psychological illnesses (e.g., PTSD), previously known associations (e.g., pulmonary diseases), and new associations (e.g., certain diagnostic procedures). In conclusion, we propose a novel HIV risk estimation method that builds on existing clinical data. It incorporates a wide range of features, leading to a model that is independent of expert opinions. It supports providers in making informed decisions in the point-of-care diagnostics process by estimating a patient's HIV risk.

## 1. Introduction

HIV and AIDS are an ongoing global pandemic. For the year 2020, the Joint United Nations Programme on HIV/AIDS (UNAIDS) reported an estimated 38 million HIV-positive people worldwide, with 1.7 million new infections and 690,000 deaths in 2019 alone (1).

In the face of these numbers, it is evident that significant efforts are needed to combat the spread of HIV. One of the measures needed to achieve this goal is the extensive use of diagnostic methods to detect infections as early as possible (2). The earlier an HIV infection is detected, the better the outcome (3), the fewer infections can occur (4), and the less of a financial burden on the healthcare system develops from this infection (5). One major challenge in the wide-spread provision of diagnostic procedures are the high costs of providing point-of-care (POC) diagnostics to every patient (6).

Machine learning (ML) and artificial intelligence (AI) methods have recently been used to estimate the HIV risk of individual patients to aid providers in bringing diagnostic services to their patients (7). These methods offer the benefit of providing fast and objective estimations of a patient's risk of getting infected with HIV or, similarly, the potential of this patient benefitting from pre-exposure prophylaxis (PrEP).

Many studies are concerned with particular groups of patients (such as women, men who have sex with men, or drug users) or contexts other than clinical care. In contrast, the present study is concerned with general-population HIV risk estimation based on the patients' clinical history. Notable studies in this field include the following. Ridgway et al. developed a predictive analytics system based on information available when the patient is first triaged. The system prompts HIV prevention counselors to discuss PrEP usage with patients predicted to meet the official criteria for PrEP usage (8). Krakower et al. evaluated various ML models on large clinical datasets, with LASSO, a form of linear regression, outperforming other methods (9). Marcus et al. also built upon LASSO (10). They showed that, in addition to sexual orientation and sexually transmittable diseases, other features in clinical data could improve the method's performance. Ahlstrom et al. used national registry data from Denmark to train various ML models (11). In their study, random forests achieved the best results.

One common point of discussion in these studies are the biases and disparities contained in the corresponding models. This reflects the bias inherent in HIV prevention (7) and should be considered when approaching patients with the subject of HIV. To cite Lazarus, "patients do not like to feel that they are being singled out" (2). For example, an ML prediction model that decides that a person is HIV-positive based on one single socio-demographic factor would be problematic.

Hence, the features included in risk estimation models play a vital role and need to be chosen objectively. Different approaches to the feature selection process have been used, namely literature research (10), the consultation of experts or guidelines (8–10), or the use of results from previous studies (11). To the best of our knowledge, this is the first study to analyze HIV risk estimation based on a patient's previous clinical records while including a wide range of features without relying on expert knowledge.

The role of expert knowledge in ML and AI-based HIV prevention has previously been described (7). While these features sets lead to powerful predictive models, it is unclear if features previously not considered in these studies might carry predictive power with respect to HIV risk. There could, unknowingly, exist features with high predictive power. The use of these features in HIV risk estimation could reduce the disparities in PrEP usage and PrEP indication (12) and the biases and disparities discovered in the models mentioned above.

This approach of taking a limited set of features is in part necessary to work with many of the ML and AI methods that are widely used. These expect data in the form of a table. Including all potential features in such a table is infeasible due to their high number, which reaches into the hundreds of thousands.

In data mining, a field that is concerned with detecting patterns in large amounts of data, association rules are often used to detect rules in heterogeneous data. These rules describe sets of items that frequently occur together. While initially developed to analyze large and heterogeneous datasets (13), such rules can also be used as a method of classification, a field known as associative classification (14).

We propose to use association rules to detect objective rules that describe a patient's HIV risk. As these rules are derived from the set of all available features, they allow providers to analyze the biases in clinical data. This allows them to use the rules as an objective first evaluation of a patient's HIV risk, solely based on their clinical history. We envision this evaluation as being implemented in existing clinical information systems, making their wide-spread use automated and readily available. This makes the automated assessment a cost-effective first line of a POC diagnostics method (assuming that an electronic health record system is already available), where the automated HIV risk estimation informs the next diagnostic steps such as an HIV test or PrEP counseling. Additionally, the rule based nature allows providers to understand the algorithm's decision and the evidence recorded in the data set that resulted in this decision. As an HIV infection is often met with stigmatization, knowledge and careful consideration of ML model's inner workings will be important factors if automated algorithms are to be used in real-world contexts.

Apart from the use as a risk estimation method, another important aspect is which features are identified as beneficial in the estimation. This sheds light on which clinical data could be considered when assessing HIV risk factors.

## 2. Materials and Methods

### 2.1. Data

We used data from the MIMIC-IV project, version 0.4 (15). MIMIC-IV is a publicly available dataset with around 525,000 hospital stays that included an intensive care unit or emergency department stay, containing various features. To model a clinical dataset as it would be available in general contexts (i.e., not only in an intensive care unit or emergency department), we limited the types of features used in this study. A list of all feature types used in this study can be found in Table 1. We included 171,657 different features *x* in this study. In this context, a feature *x* can be any item that was recorded during the clinical stay. Examples include diagnoses, procedures, socio-demographic factors like gender, insurance type, and marital status, as well as the services and wards that the patient has visited. There was no further feature selection process. This high number of features reflects the complexity of both clinical care and HIV symptoms.

**Table 1:** A list of all feature types used in this study with a description and the number of features of that type that are available in MIMIC-IV.

**Feature type**	**Description**	**Number of features**
Diagnosis	Diagnoses as ICD codes	86,751
DRG	Case group	2,059
Ethnicity	Patient's ethnicity	8
Gender	Binary: male/female	2
Insurance type	Medicare, Medicaid, Other	3
Language	Binary: English/other	2
Marital status	Single, married, divorced, widowed, missing	5
Procedure	Procedures as ICD codes	82,763
Service	Clinical services like medical, psychological	21
Ward	Clinical wards like emergency department, surgery	43
Overall		171,657

*DRG, Diagnosis-Related Group; ICD, International Classification of Diseases*.

The goal is to estimate the HIV risk of patients based on their clinical history. Thus, only patients with at least two stays were included. The HIV infection must not have occurred in the first stay of the patient. This resulted in 85,216 study patients, out of which 38,765 were male and 46,451 female. These patients account for 349,111 hospital stays. We determined all HIV-positive patients by filtering for the patients that have one of the twelve HIV diagnoses in MIMIC-IV, see Table 2. No AIDS diagnosis was recorded in MIMIC-IV. As the HIV-positive patients will eventually contract HIV during the time recorded in MIMIC-IV, these patients are considered to have a high risk of contracting HIV prior to the infection. This choice of 12 diagnoses resulted in 521 HIV-positive patients, out of which 411 were male, and 110 were female.

**Table 2:** A list of all HIV diagnoses recorded in MIMIC-IV.

**Diagnosis**	**ICD version**	**ICD code**
HIV disease	9	042
HIV, type 2	9	079.53
Asymptomatic HIV infection status	9	V08
HIV disease	10	B20
HIV, type 2 as the cause of diseases classified elsewhere	10	B97.35
HIV disease complicating pregnancy, first trimester	10	O98.711
HIV disease complicating pregnancy, second trimester	10	O98.712
HIV disease complicating pregnancy, third trimester	10	O98.713
HIV disease complicating pregnancy, unspecified trimester	10	O98.719
HIV disease complicating childbirth	10	O98.72
HIV disease complicating the puerperium	10	O98.73
Asymptomatic HIV infection status	10	Z21

*ICD, International Classification of Diseases*.

### 2.2. Incidence Rate Ratio

The association between the features *x* and HIV was measured using the incidence rate ratio (IRR). It is commonly used in epidemiology as a causal metric that measures how a risk factor influences an outcome. An IRR of 1.0 denotes no association, while higher or lower IRRs denote a positive or negative association, respectively.

As a fictional example, suppose we ran a study with ten patients. Five of the patients were smokers, the other five non-smokers. The smokers spent 60 life-years in the study, and three out of the five smokers were infected with HIV during their time in the study. After their infection, the patients' lifetime was not included in the 60 life-years. Among the non-smokers, there were 50 life-years recorded in the study, with two patients being infected within the study duration. These two patients were also excluded from the life-years calculation from this point on. In summary, the incidence rate for HIV was 3/60 person-years among smokers and 2/50 person-years among non-smokers. The incidence rate ratio for smoking and HIV is then calculated as the ratio of the incidence rates, i.e.,


(1) $$\begin{array}{l} \text{IRR}\left(\text{Smoking}\right)=\frac{3/60\text{person-years}}{2/50\text{person-years}}=1.25. \end{array}$$

This indicates that there is a slight positive association between smoking and an HIV infection in this fictional example.

While the present study is concerned with association and not causality, we use IRRs to measure an association between different features and an HIV infection. They capture temporal effects and measure the strength of an association, which allows us to assess the predictive value of a feature in terms of HIV risk.

### 2.3. Rule Mining

Let the dataset be denoted by D. It is a collection of patients, each of which is a collection of hospital stays. Each hospital stay is modeled as a collection of feature values and a timestamp denoting the date and time of the admission. Clinical documentation might not occur at the same time as some event occurs. This is why we assume all clinical items in one stay to occur at the time of admission. The difference in admission times is expected to be larger than the different event times in one stay, i.e., the intra-stay time differences are expected to be negligible compared to the inter-stay time differences of each patient.

**Figure 1:**
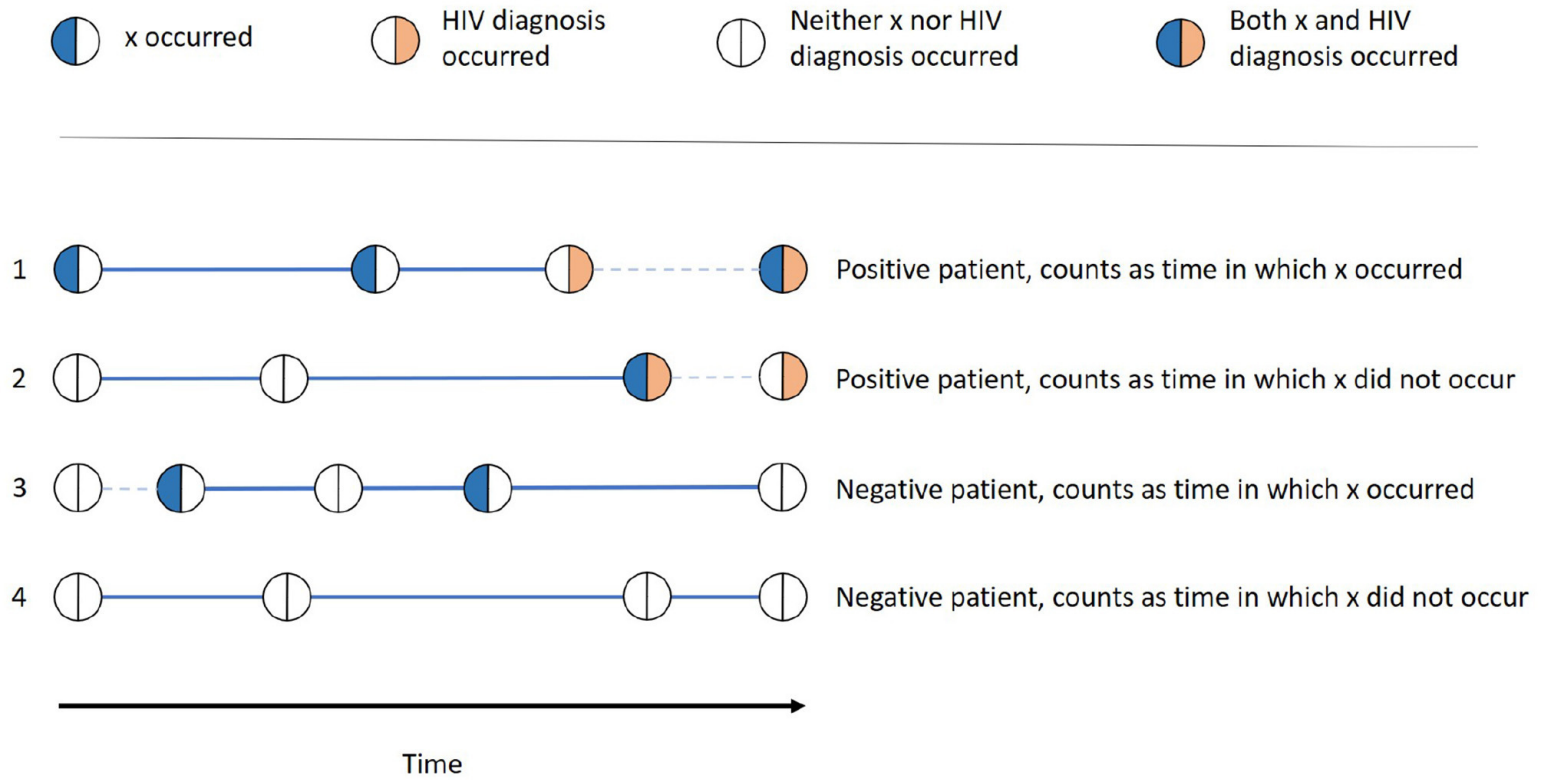
Examples of all different possibilities of how one patient can be categorized into the groups x occurred, x did not occur, positive, and negative. The thick lines denote the time in which x occurred resp. did not occur, while the dashed lines denote the time that does not contribute to this patients time span.

As the IRR is aggregated from values calculated for every single patient *P* and every single feature *x*, we can look at one patient and one feature at a time. The different variations we can find in the history of that patient are depicted in Figure 1. We first consider whether the patient was HIV-positive, i.e., if one of the twelve diagnoses in Table 2 occurred. If this is the case (1 and 2 in Figure 1), we denote the first stay in which the outcome occurred as the "last stay of interest" and count that patient as HIV-positive. If this is not the case (3 and 4 in Figure 1), we take the actual last stay recorded for the patient as the "last stay of interest" and count that patient as HIV-negative. This "last stay of interest" is the point in time when the patient was diagnosed with HIV, after which we do not need to estimate the HIV risk anymore, or when there is no further information on the patient available.

Whether *x* occurred for the patient is determined by whether the patient had a stay with the feature value *x* before the last stay of interest. If this is the case (1 and 3 in Figure 1), the patient is counted as one patient for whom *x* occurred and the time between that first occurrence and the last stay of interest is the time in which *x* occurred of that patient. This time span is denoted as a thick line in Figure 1. For the patients for which there is no stay with *x* before the last stay of interest (2 and 4 in Figure 1), the time between the patient's first overall stay and the last stay of interest is the time in which *x* did not occur for that patient, also denoted as a thick line in Figure 1.

For each patient, *x* either occurred (thus only contributing to time in which *x* occurred) or did not occur (thus only contributing to time in which *x* did not occur) If there are stays before the occurrence of *x* is recorded, the time until the first occurrence is not counted as time in which *x* did not occur. This is because the occurrence of *x* might have already been there, but it was simply not recorded or known, e.g., in the case of an unknown diagnosis.

After these calculations have been done for every patient *P*, the corresponding counts and times are summed up. The incidence rates are then calculated as


(2) $${\text{IR}}_{x}=\frac{\left|\{P\in D\;|x\text{ occurred and }P\text{ is HIV-positive }\}\right|}{\sum_{P\in D}\text{time before infection}},$$

and accordingly


(3) $${\text{IR}}_{\neg x}=\frac{\left|\{P\in \;D\;|x\text{ did not occur, but }P\text{ is HIV-positive}\}\right|}{\sum_{P\in D}\text{time before infection}}.$$

The IRR is then the ratio of the incidence rates of patients for which *x* occurred vs. those for which *x* did not occur, i.e.,


(4) $$\begin{array}{l} \text{IRR}\left(x\right)=\frac{IR_{x}}{IR_{\neg x}}. \end{array}$$

A statistical test for "IRR(*x*) ≠ 1" can be used to ensure that the discovered IRRs are statistically significant. The presented approach uses the approximately normal distribution of the natural logarithm of the IRR (16). A Wald test (17) is executed for each discovered rule based on this distribution, resulting in a two-sided *p*-value. The maximally allowed *p*-value is configurable. Bonferroni correction can be applied to accommodate for multiple hypothesis testing (18). All rules whose *p*-value lie above a configured value are filtered out. Additionally, all rules with less than a configurable minimum number of HIV-positive and HIV-negative patients are filtered out. This is done to ensure that the patterns are not caused by a few patients with extremely short periods between the occurrence of *x* and an HIV diagnosis, for example.

### 2.4. Risk Estimation

The rules are mined from the dataset D as described above. Given a new patient, these rules are used to estimate the patient's HIV risk.

First, all the rules are evaluated for the new patient. Only rules that apply to the patient (i.e., rules for which the feature *x* occurred in the patient's clinical history) are kept. Second, the mean IRR of all applying rules is calculated.

This mean IRR is then used as the HIV risk score. A threshold has to be set to determine which patients are HIV-positive. Risk scores above that threshold define high-risk patients. The choice of a threshold is based on clinical requirements. With lower thresholds, more patients will be declared as high risk patients, leading to more false-positive predictions, but also more true positive predictions. Higher thresholds lead to more true and false-negative predictions. The use of a threshold is thus a trade-off between false negative and false positive predictions.

### 2.5. Experiments

The data was randomly split into 20% test data and 80% training data to test and validate the method. To improve the performance in the training phase, 90% of the HIV-negative patients have been randomly filtered out from the training set. This results in around 7,000 training patients, 400 out of which are HIV-positive, and around 17,000 test patients.

The following hyperparameters were used. First, the minimum number of HIV-positive and HIV-negative patients ranged from 0 to 10. For the statistical significance test, the *p*-value thresholds *p* < 1, *p* < 0.05, and *p* < 0.001 were used. In addition, the use of Bonferroni correction was configurable (as a binary yes/no decision). This results in 11·3·2 = 66 possible hyperparameter configurations.

Each experiment was repeated ten times. All experiments were evaluated via the area under the receiver operating characteristic curve (AUC), which is commonly used in binary classification. It shows the sensitivity (identified positive patients divided by all positive patients) against (one minus) the specificity (identified negative patients divided by all negative patients) for different threshold values.

We implemented the method in the programming language C#.

## 3. Results

We evaluated the experiments using the programming language R (19) and the tidyverse packages (20).

**Figure 2:**
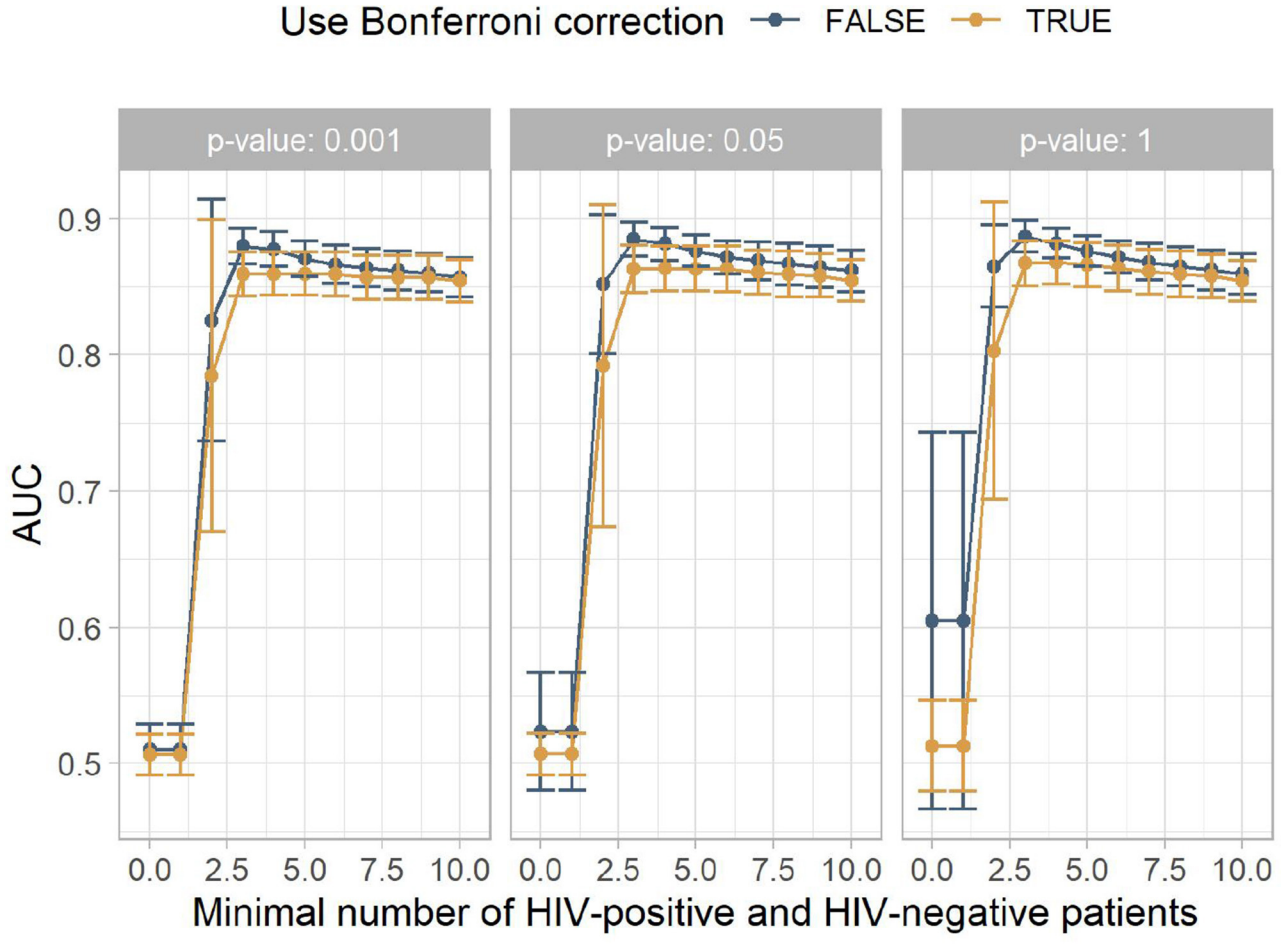
The mean area under the receiver operating characteristic curve (AUC) for the different experiment configurations. The x-axis shows the minimum number of HIV-positive and HIV-negative patients, the facets show different *p*-value thresholds, the color indicates if Bonferroni correction was used, and the y-axis shows the mean AUC achieved by this configuration, with the error bars indicating plus/minus one standard deviation.

The results are summarized in Figure 2. Statistical significance tests did not improve the classification performance. The best mean results were achieved with *p* < 1 and no Bonferroni correction. The minimum number of HIV-positive and HIV-negative patients, which is needed for a rule to be included in the model to ensure that the rule is not caused by outliers, had more impact on the AUC. Up to around a minimum number of HIV-positive and HIV-negative patients of four, this filter heavily increases the classification performance, leading to the best AUC being 0.88. This is comparable to other studies that build on electronic health records (9, 10). From four onwards, the mean performance steadily declines.

**Figure 3:**
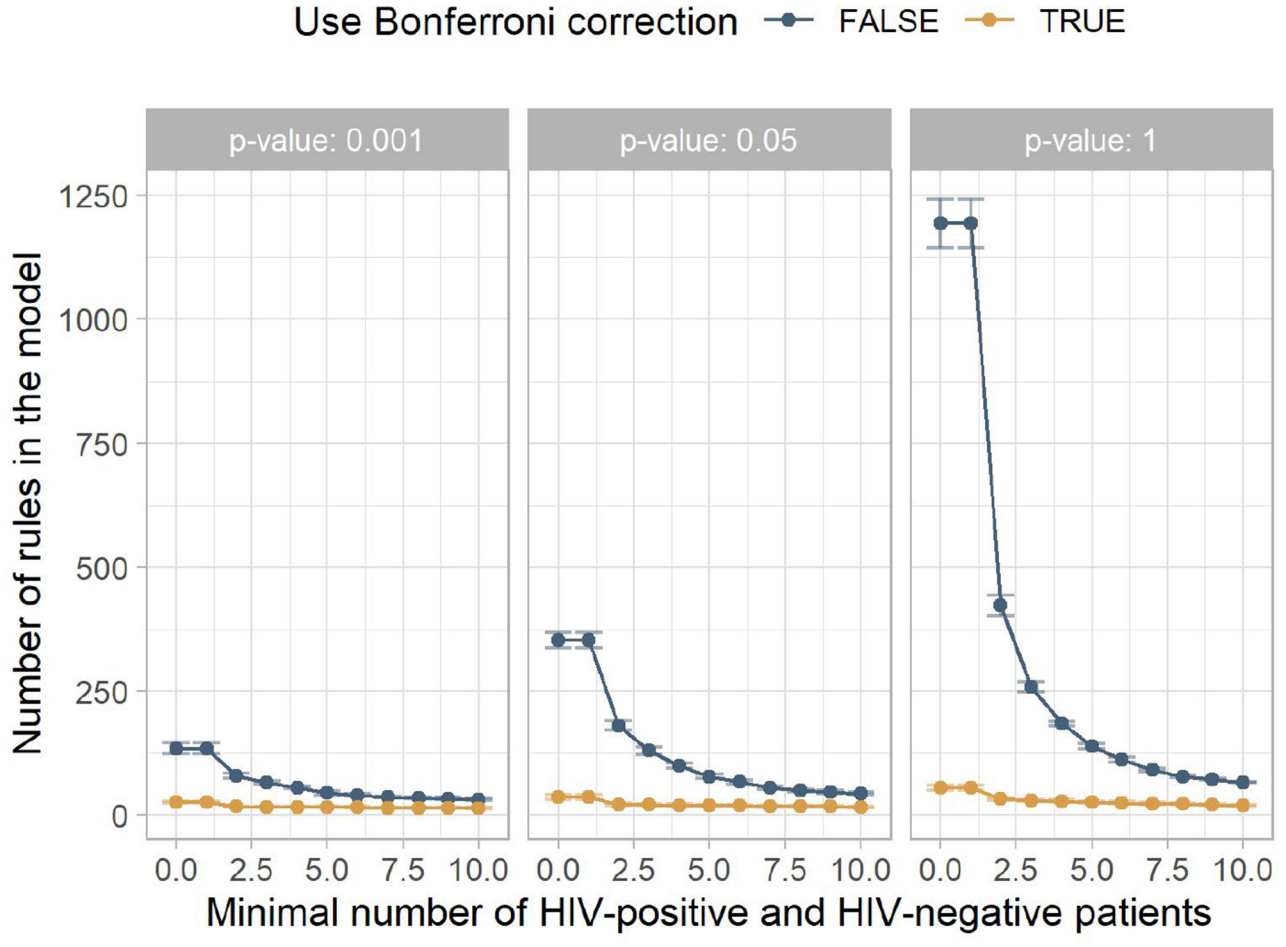
The mean number of rules in the model for the different experiment configurations. The x-axis shows the minimum number of HIV-positive and HIV-negative patients, the facets show different *p*-value thresholds, the color indicates if Bonferroni correction was used, and the y-axis shows the mean number of rules returned by this configuration, with the error bars indicating plus/minus one standard deviation.

Figure 3 shows the mean number of rules for the models contained in Figure 2. It indicates that low minimum numbers of HIV-positive and HIV-negative patients lead to many rules which do not contribute to a correct classification. The larger this hyperparameter is chosen, the fewer misguiding rules are contained in the model, increasing the AUC. At higher minimum numbers of HIV-positive and HIV-negative patients, this filtering includes rules with higher predictive performance, which slowly reduces the AUC.

**Figure 4:**
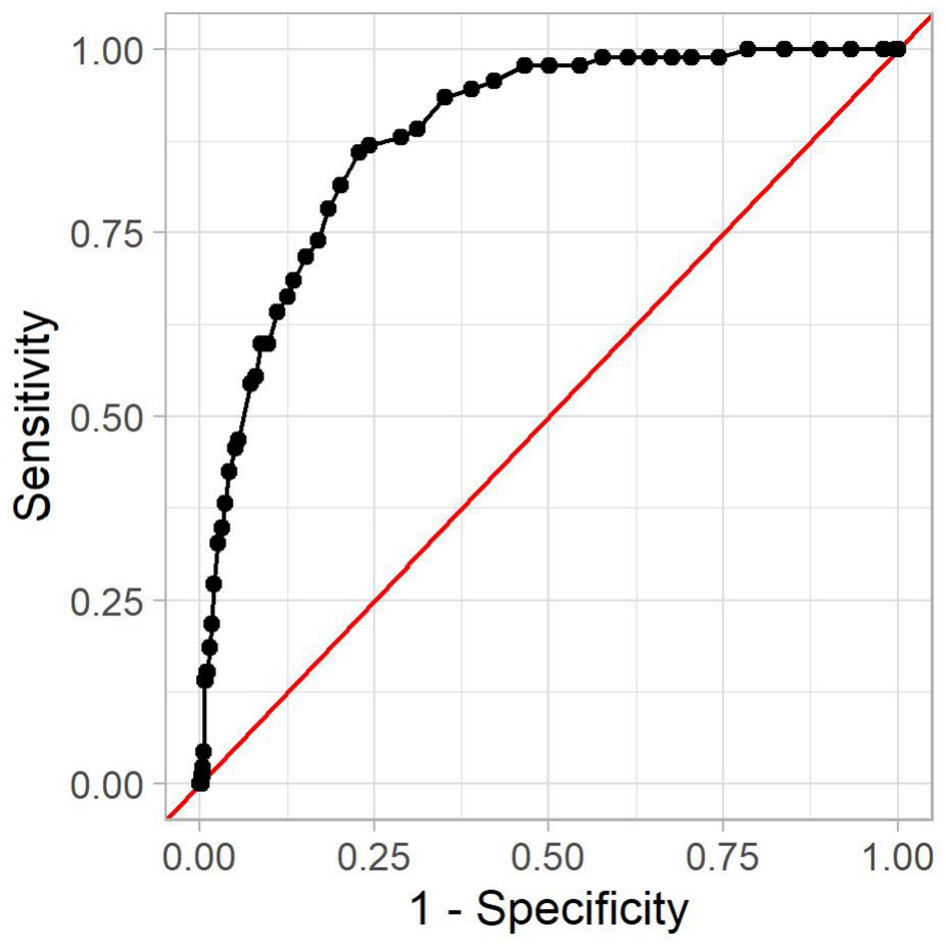
The receiver operating characteristic curve for the reference model. The x-axis shows (one minus) the specificity, and the y-axis shows the sensitivity. Every dot is one possible threshold value. The red line denotes the curve of a random classifier (i.e., a coin toss).

In the following, we analyze one model in more detail. It was created using a minimum number of HIV-positive and HIV-negative patients of four, no Bonferroni correction, and a *p*-value threshold of 0.001. As more strict statistical significance tests filter out more rules, this model contains few rules, namely 53, with 14 positive associations. This model achieved a good AUC of 0.88. The corresponding receiver operating characteristic curve can be seen in Figure 4.

A threshold value is used to decide which IRR is high enough for a patient to be considered positive. The threshold value affects sensitivity and specificity of the prediction model. We further examine the model with a threshold of 0.66. It leads to an accuracy of 54%, a sensitivity of 98%, and a specificity of 53%, with 2 out of 92 HIV-positive patients in the test set being misclassified as negative. Out of the 16,983 HIV-negative patients in the test set, 7,907 were incorrectly classified as positive. This means that if the proposed method flags a patient as high-risk, the probability that this patient is indeed high-risk is 1.1%. This is due to the relatively low prevalence of HIV infections among the population. While almost all high-risk patients are detected, this also includes many low-risk patients. In contrast, a patient flagged as low-risk will indeed be low-risk in almost 100% of all cases. Other threshold values lead to different performance metrics, which is why the optimal threshold value depends on the usage context at hand. This is mostly a compromise between detecting as many HIV-positive patients as possible and avoiding troubling patients with HIV tests.

There are hyperparameter configurations that lead to slightly higher AUCs (improvement < 0.01 percentage points) or fewer rules in the model, but this configuration strikes a good balance between performance and size.

We chose an interpretable model in order to be able to analyze the underlying associations explicitly. In addition to the good performance in HIV risk estimation, the model also sheds light on features that might hint at an HIV infection but could be overlooked by providers because of the diffuse manifestations of HIV. Note that these rules only apply to the patient population of the hospital in which the dataset was generated. However, many of the rules reproduce previous studies on HIV.

The model consists of 53 rules, including 14 positive rules. One pattern spanning several rules is drug abuse (IRR 1.99–16.41), which also contains the rule with the highest IRR. Drug abuse has previously been established as a risk factor for HIV (21). Another high rule is the diagnosis of PTSD (4.33). Other psychological issues can be found in the negative rules, namely anxiety (0.13) and depressive disorder (0.32). Other positive association rules reflect well-known gender and racial disparities in the United States (7, 22), like the increased prevalence of HIV in males (2.72) as well as in the black population (1.57).

Similarly, social factors play a role, for example in the form of Medicaid insurance (1.96), which could be due to confounding factors. This includes the patient's marital status, as being single is highly associated with an HIV infection (3.08). To the best of our knowledge, the association between marital status and HIV infection has not been studied in a general United States population, but studies on HIV among military personnel (23) and HIV mortality in the United States (24) suggest that there is a positive association between being single and being infected with HIV, possibly due to having more sexual contacts and lower social integration (24).

Another pattern are diagnostic procedures of the lung (1.73–2.58), possibly hinting at pulmonary health problems caused by HIV (25). Skeletal x-rays of thigh, knee, and lower leg (5.58) and of wrist and hand (5.78) are highly associated with HIV. These two rules might hint at the higher risk of falling for HIV-positive patients (26). The remaining rules are vein punctures (1.75) and spinal taps (4.25). Both are common diagnostic procedures with no extraordinary connection to HIV.

Many of the 39 negative rules complement positive rules, such as being female (0.22), being white (0.46), being married (0.18) or widowed (0.19), and being insured under Medicare (0.69) or "other" (0.38).

One more aspect that occurs in various rules is the clinical services (0.11–0.55) and wards (0.10–0.50) that a patient has visited. Such associations have previously been established (27). As the dataset used in this study is focused on intensive care unit and emergency department stays, the exact findings differ. However, it is evident from the rules that the information which clinical services and ward a patient has previously visited can be used when estimating their HIV risk. Other negative rules were previously studied, e.g. the use of anticoagulants (0.13) in HIV prevention (28). The negative rules also contain some surprises. While most drug abuse rules are positive associations with HIV, "Personal history of tobacco use" (0.12) and "Tobacco use disorder" (0.23) are negatively associated with HIV.

The list of all rules in the model can be found in the Supplementary Materials.

## 4. Discussion

The first of the United Nations' 95-95-95 goals is that 95% of all HIV-positive persons should know of their HIV infection. To aid providers in targeting patients with high HIV risk, we proposed an HIV risk estimation method based on ML techniques, namely associative classification based on the IRR. As it is based solely on clinical data, it can automatically run in the background and provide a first assessment directly in the hospital or clinic. It can be used as a first-line POC diagnostics method for large populations to estimate an individual's HIV risk before assessing the next steps like an HIV test or PrEP counseling. The goal is not to automatically prompt an HIV test or PrEP intake based on the algorithm's decision, but to inform providers to take these tools into consideration. With a sensitivity of 98%, the proposed method is very good at detecting HIV-positive patients, which can then be targeted by providers.

In contrast to existing methods, the resulting model contains rules that are objectively discovered from clinical data and independent of expert knowledge, allowing providers to consider features that have previously not been used in HIV prevention, like previous diagnoses or clinical services. Even for experts, the amount of features and their association to HIV is too much to memorize, so such rules can clarify which features can be used to obtain a usable HIV risk estimation model. As the model takes its information from clinical data, it is also independent of sensitive information like sexual orientation and transgender status. Being gay or transgender, for example, is in many parts of the world punishable. Talking about sexual and gender issues needs trust between provider and patient, which is not always given. The proposed method lets providers use more readily available information about their patients. This information comes in the form of a risk score that can be binarized, but the model offers more guidance than that. As it is composed of rules that may apply to a given patient, the provider can get an overview of which HIV-associated features have been recorded in the patient's clinical history. If the provider deems these features suspicious, they can use that information to prescribe HIV testing independent of the risk score determined by the model.

Apart from this advantage of objectivity regarding the choice of features, the proposed method has some other major advantages. First, its interpretability has benefits for all stakeholders. While providers can understand the algorithm's decision and question its correctness, patients can see why providers approach them with the sensitive subject of HIV. For researchers, the interpretability leads to insights on the underlying dataset in the form of if-then rules. We have seen that several rules in the model correspond to previous studies on HIV, while others might hint at new associations. Second, the method builds on data that comes from the electronic health records that are available at the point of care, which is a hospital or clinic in most cases. As all information that goes into the model is available, no further equipment is needed. Access to the underlying database suffices to train the model and apply it to patients' data. This makes the proposed method cost-effective for first screening approaches. Third, the proposed method outperforms other methods that build upon features from electronic health record data (AUCs 0.75–0.86) (9, 10, 29). Note that a direct comparison of these methods is difficult due to different underlying datasets. Future work is needed to compare the different models further. Another point of comparison are manual HIV risk score methods. One example that is often used in the United States is the Denver HIV Risk Score (30). Similar to Machine Learning methods, 48 candidate variables have been determined based on expert knowledge. Using logistic regression, a pruned model with eight variables was constructed. In various validation studies in the United States and Canada, the AUCs ranged from 0.75 to 0.80 (30–33). The proposed method thus outperforms the Denver HIV Risk Score. Lastly, the proposed method is able to incorporate thousands of features. While other approaches build upon features from electronic health record data use between 81 and 1,583 features (9, 10, 29) and the Denver HIV Risk Score uses 48 features (30) during training, our proposed approach incorporates 171,657 features into the model training. As this necessarily includes features that have not been considered before, this might be one reason for the performance improvement.

The proposed method also has some limitations. First, the dataset used in this study, MIMIC-IV, has a particular population, as it is focused on intensive care unit and emergency department stays. More general clinical datasets will lead to different rule sets. However, the model discussed here had a good performance, despite the shortcomings of the underlying dataset. Second, the model itself is dependent on the underlying clinical datasets. Other clinical datasets (whether on a hospital, hospital network, or national level) will lead to different rule sets as they have other coding guidelines and clinical practices. Therefore, the generated model and the corresponding rules are only valid in the hospital in which the dataset was generated. The differences in rule sets from different context themselves will be an interesting object of study. While rule sets from different hospitals in the same city and thus with similar patient populations might hint at differences in the care provided, rule sets from different regions might give us insight into differences in the distribution of HIV infections among the populations. The proposed method, however, is usable in general contexts. While it will lead to different models, it can still be used to objectively analyze large amounts of features for predictive qualities with respect to HIV.

There are several potential directions for future work. First, the comparison of rule sets created from different databases will be an interesting point of study. As the rules reflect the underlying patient population, regional or national differences in the rule sets can hint at differences in the population, HIV prevention strategies, and differences in the healthcare systems. Second, personalization methods as described by Valente et al. (34) might improve the predictive performance, while also bringing reliability estimations into the prediction model. Third, the method might be useful in the prediction of other diagnoses or in other tasks from different fields altogether, especially if thousands of features are to be considered. More research on applications of the proposed method will allow us to analyze its general usability. Lastly, different models for HIV prediction based on electronic health records exists. Further analysis of the strengths and weaknesses of the approaches as well as their differences and similarities are expected to shed light on further improvements. Approaches like *bagging* (35) could lead to better predictive performance by combining the models into one aggregate model. This might come at the cost of reduced interpretability. Further work is needed to study such approaches.

## 5. Conclusion

We proposed a novel HIV risk estimation method. It builds upon large amounts of features to find those that can be used to estimate a patient's HIV risk from their clinical history. It produces a good predictive model that can be used as a first-line POC diagnostics method for large and general populations to inform further steps. The rules detected in this model are easy to interpret. Many study results on HIV were reproduced. Some rules were surprising and might be used as a starting point for future clinical studies.

## Data Availability Statement

Publicly available datasets were analyzed in this study. This data can be found at: https://physionet.org/content/mimiciv/0.4/.

## Author Contributions

OH: concept, programming, analysis of the results, and writing of the manuscript. AM and ER: substantial revision of the work. All authors have approved the submitted version and take responsibility for the scientific integrity of the work.

## Funding

This project was funded by the Bavarian State Ministry of Science and the Arts and coordinated by the Bavarian Research Institute for Digital Transformation (bidt) and supported by the Bavarian Academic Forum (BayWISS) Doctoral Consortium Health Research.

## Conflict of Interest

The authors declare that the research was conducted in the absence of any commercial or financial relationships that could be construed as a potential conflict of interest.

## Publisher's Note

All claims expressed in this article are solely those of the authors and do not necessarily represent those of their affiliated organizations, or those of the publisher, the editors and the reviewers. Any product that may be evaluated in this article, or claim that may be made by its manufacturer, is not guaranteed or endorsed by the publisher.

## Supplementary Material

Supplementary Table 1 The rules in the final model.
